# Supplementary material for: Optical control of exciton spin dynamics in layered metal halide perovskites via polaronic state formation
Source: Nat Commun. 2022 Jun 9;13:3320. doi: 10.1038/s41467-022-30953-w (PMC9184503; doi:10.1038/s41467-022-30953-w)
Supplement: Supplementary file 1 — Supplementary Information [file 41467_2022_30953_MOESM1_ESM.docx]

**Supplementary Information**

Optical control of exciton spin dynamics in layered metal halide perovskites via polaronic state formation

Sean A. Bourelle^1†^, Franco V. A. Camargo^2†^, Soumen Ghosh^3†^, Timo Neumann^1,4^, Tim W. J. van de Goor^1^, Ravichandran Shivanna^1,5^, Thomas Winkler^1,6^, Giulio Cerullo^2,3^*, Felix Deschler^4,♦^*

^†^ These authors contributed equally.

1 Cavendish Laboratory, University of Cambridge, J J Thomson Avenue, Cambridge CB3 0HE, UK

2 Istituto di Fotonica e Nanotecnologie-CNR, Piazza Leonardo da Vinci 32, 20133 Milano, Italy

3 Dipartimento di Fisica, Politecnico di Milano, Piazza Leonardo da Vinci 32, 20133 Milano, Italy

4 Walter-Schottky-Institute, Physics Department, Technical University Munich, Am Coulombwall 4, Garching, Germany

5 School of Advanced Sciences, Vellore Institute of Technology, Chennai, India

6 Department of Physics and Astronomy, Aarhus University, 8000 Aarhus C, Denmark

♦ Current address: Physikalisch-Chemisches Institut, Universität Heidelberg, Im Neuenheimer Feld 229, 69120 Heidelberg, Germany

* Correspondence should be sent to G.C. (giulio.cerullo@polimi.it) and F.D. (deschler@uni-heidelberg.de)

**Supplementary Note 1: Mechanisms of spin relaxation**

Faraday rotation measurements are used to track the depolarisation of exciton total angular momentum states following excitation with circularly polarised light. Optical excitation using circularly polarized light generates excitonic states with polarized total angular momentum |±1⟩^1^. These states then depolarize via lattice and exciton interactions, with only the former being relevant under natural sunlight illumination levels. Therefore, we perform all measurements at low fluences before many body interactions play a significant role (see Supplementary Figure 2 for fluence dependence data). While the optically excited total angular momentum states are not spin pure^2^, the spin ratios within each total angular momentum state are constant. Thus, exciton spin depolarization and the depolarisation of total angular momentum projection occur simultaneously and are tracked together by Faraday rotation. Depolarisation often falls into a motional narrowing, or scattering induced class, as determined by temperature dependence. Examples of these relaxation mechanisms are discussed below.

1. **D’yakonov-Perel spin relaxation**

For systems without inversion symmetry, the spin degeneracy is lifted for $K\neq0$ such that carriers of opposite spins have different energy when moving at the same $K$. This difference in energy arises due to the opposite coupling between electron spin and the effective magnetic field that is generated by the motion of the charge carrier through an electric potential:

| $H_{SO}=g\mu\vec{B}_{eff}.\vec{S}$ | **(1)** |
| --- | --- |

where,

| $\vec{B}_{eff}\propto\vec{\nabla}V\times\vec{P}$ | **(2)** |
| --- | --- |

relates the magnitude of the effective magnetic field to the magnitude of the electron momentum $\vec{P}$, in the potential gradient $\vec{\nabla}V$.If uninterrupted, carrier spin processes around this magnetic field at a momentum dependent rate such that spin polarisation is quickly lost.

Thus, D’yakonov-Perel spin relaxation is of the motional narrowing class whereby phonon scattering at a rate, $\Gamma$, suppresses spin precession and increases the spin depolarisation lifetime:

| $\tau_{S}\propto\frac{\Gamma}{{Ω\left( \vec{K} \right)}^{2}}$ | **(3)** |
| --- | --- |

where $Ω\left( \vec{K} \right)$ describes the rate of precession and is dependent on the exciton center of mass wave vector $\vec{K}$. The validity of defining an exciton ***K***-vector has been established by recent charge transport measurements that demonstrated band-like transport^3^. The scattering rate, $\Gamma$, has a well-defined temperature dependence as determined by the temperature dependence of the various scattering mechanisms, such as phonon scattering,$\Gamma_{P}$, or carrier-carrier scattering $\Gamma_{e}$:

| $\Gamma=\alpha\Gamma_{P}+\beta\Gamma_{e}+\ldots$ | **(4)** |
| --- | --- |

where $\alpha$ and $\beta$ are coefficients that describe the relative contributions of each scattering mechanism (Matthiessen’s Rule). We note that scatting rates for: acoustic phonon (deformation potential) scattering scale as $T^{3/2}$; acoustic phonon (piezoelectric) scattering scale as $T^{1/2}$; ionised impurity scattering scale as $T^{-3/2}$; and carrier-carrier scattering shows little temperature dependence^4^.

When $|K|$ has a strong energy dependence, the precession term $Ω\left( \vec{K} \right)$will also scale with temperature and exciton energy. An increase in pump photon energy adds heat to the system which increases electron-phonon scattering. However, the hot electrons typically have a greater kinetic energy, lie higher in $K$-space, and thus experience a larger effective magnetic field around which the spin precesses. The total spin relaxation will therefore depend on the balance between faster precession and increased scattering. As $\vec{B}_{eff}$ (and consequently $Ω\left( \vec{K} \right)$) depend on the potential gradient, $\vec{\nabla}V$, which originates from lattice asymmetry, changes in the lattice configuration can lead to changes in the rate of precession.

1. **Exchange driven spin relaxation**

For excitonic systems, Maialle, Silva and Sham (MSS) derived the form of spin relaxation due to electron-hole exchange within the exciton quasiparticle. As with the D’yakonov-Perel mechanism, this belongs to the motional narrowing class—spin relaxation occurs *between* the momentum scattering events:

| $\tau_{S}\propto\frac{\Gamma}{{Ω\left( \vec{K} \right)}^{2}}$ | **(5)** |
| --- | --- |

where the rate of precession $Ω\left( \vec{K} \right)$ around an effective magnetic field is due to the off-diagonal terms within the long-range exchange Hamiltonian and is linearly dependent on the exciton’s average center of mass wave vector, $|K|$. $\Gamma$ is the momentum scattering rate, as described above.

Elaborating on points from the previous section: energy follows a linear dependence on T (when averaging over the Fermi-Dirac distribution) so, when assuming a parabolic dispersion, $|K| \propto\sqrt{E}$. However, the precession term depends on the average center of mass wavevector, which can remain independent of, or weakly dependent on T. As such, in the case of temperature independent $\bar{|K|}$

| $\tau_{S}\propto\Gamma(T)$ | **(6)** |
| --- | --- |

and the spin relaxation time scales linearly with the momentum scattering rate.

This matches our experimental data **under resonant pumping**, where the scattering rate $\Gamma(T)$ is obtained from the full width half maximum of the photoluminescence spectrum for different temperatures (see Supplementary Figure S3 and following discussion). It is worth noting that the scattering rate has previously been reported to scale with temperature as ${\Gamma\propto T}^{3/2}$ within both 2D and 3D hybrid-perovskites^5–7^. This differs from our observation of ${\Gamma\propto T}^{5/2}$, and may be attributed to the phase change beyond 240K as shown in Supplementary Figure S3.

1. **Elliot-Yafet spin relaxation**

The Elliot-Yafet (EY) spin relaxation differs from the previous two in that spin depolarisation occurs via momentum scattering events, and the spin depolarisation lifetime can be expressed as:

| $\tau_{S}\propto\frac{1}{\Gamma\chi^{2}}$ | **(7)** |
| --- | --- |

where $\chi$ describes the spin-orbit coupling (SOC) induced spin-mixing and $\Gamma$ is the momentum scattering rate, as defined above. $\chi$ depends on the average energy of the photoexcited states as this determines the average change in momentum between scattering events, and hence the probability of flipping spin. As above, the average energy scales linearly with temperature, thus:

| $\tau_{S}\propto\frac{1}{\Gamma T^{2}}$ | **(8)** |
| --- | --- |

From our measurement of the PL linewidth $\Gamma$ scales as $T^{2.5}$ between 70K and 230K. Thus, $\tau_{S}\propto$ $T^{-4.5}$ for the EY mechanism.

1. **BAP mechanism**

As with the MSS mechanism, the BAP mechanism is due to the long-range exchange interaction between an electron and a hole. However, BAP focusses on the case of doped samples, where excitonic states are unlikely to form due to the strong screening by the majority carrier population. Therefore, BAP describes the spin flip of a free electron upon scattering with a hole via the long-range exchange interaction. As BAFAPbI_7_ is an undoped perovskite, this mechanism is not expected to play a significant role.

**Supplementary Note 2: Extraction of the oscillatory TA signal**

To extract the oscillatory component of the TA signal, we select the probe energy of maximum oscillation amplitude and fit that trace with a genetic algorithm using a combination of exponential and oscillatory components:

| $\frac{\Delta T}{T}=\sum_{n=1}^{2} A_{n}e^{-t/T_{n}}\cos(2\pi\nu_{n}t+\phi_{n})+\sum_{n=1}^{3} B_{n}e^{-t/\tau_{n}}+C$ | **(9)** |
| --- | --- |

The parameters that yielded the best fit are reported in Supplementary Table S1. The second oscillatory component was used in order to partially compensate the response function and non-exponential behaviour in the early time kinetics, and dephases much more quickly than the coherent oscillations, so no real physical meaning is attached to it. The oscillatory component is extracted from the data by subtracting the non-oscillatory and overdamped oscillatory components. This is plotted Figure 3c,d of the main text for pump energies of 2.17 eV and 2.43 eV respectivelly.

**Supplementary Note 3: Scattering modified by polaron formation and their relation to the observed change spin relaxation:**

A change in the exciton-phonon scattering rate would not change the mechanism in favour of the significantly slower spin depolarization at 77K. A decrease in scattering rate would increase the spin relaxation rate due to motional narrowing and lead to shorter lifetimes, contrary to our observation. In order to attribute the observed change in mechanism to scattering, the spin relaxation must be slowed down significantly, such that a new mechanism becomes the faster source of spin depolarisation. However, our results in the main text (and those of Todd, Riley et al.) indicate that the spin lifetime at room temperature is well below 40 ps. Therefore, while increasing the scattering rate can slow spin relaxation, it is not enough to extend lifetimes beyond 40 ps, as is required for the new mechanism (that shows inverse temperature dependence) to dominate.

Screening of the Fröhlich interaction with other phonons would reduce phonon scattering. This would decrease the spin lifetime under the motional narrowing regime, while we see an increase after polaron formation. We therefore conclude that it is not applicable.

The energy distribution of polarons indeed tends to be broader than that of excitons at low temperatures since polaron formation does require interaction with phonons. However, following excitation at 2.43 eV the excitons themselves also interact with phonons and indeed we see that a fast component of 0.2 ps, similar to that observed in the 2.17 eV experiment, remains present. The intermediate component at ~3 ps indeed seems to be due to failed crossings of the activation barrier, which is an effect of the energy distribution. Nonetheless, it seems that the predominant mechanism is the polaron formation and the change in the electron-hole overlap that this entails.

**Supplementary Table 1: Parameters of the fits in Figure 3c,d.** The amplitudes and baseline have units of percentual $\Delta T/T$, while the phases are given in radians.

| Pump | $A_{1}$ | $T_{1}$(ps) | $\nu_{1}$(ps^–1^) | $\phi_{1}$ | $A_{2}$ | $T_{2}$(ps) | $\nu_{2}$(ps^–1^) | $\phi_{2}$ | $B_{1}$ | $\tau_{1}$(ps) | $B_{2}$ | $\tau_{2}$(ps) | $B_{3}$ | $\tau_{3}$(ns) | $C$ |
| --- | --- | --- | --- | --- | --- | --- | --- | --- | --- | --- | --- | --- | --- | --- | --- |
| 2.17 | 0.054 | 2.15 | 1.52 | 4.34 | 0.463 | 0.3 | 1.04 | 2.65 | 1.76 | 0.16 | 0.32 | 6.3 | 0.54 | 4.1 | 0.11 |
| 2.43 | 0.244 | 1.24 | 1.53 | 3.79 | 0.303 | 0.63 | 0.82 | 4.02 | 6.47 | 0.35 | 0.62 | 5.1 | 0.01 | 6.2 | 0.07 |

**Supplementary Figures:**

|  |
| --- |
| **Supplementary Figure 1:** **Temperature dependent absorption spectrum.** Peak narrows as temperature is reduced from 240K (orange) through (180K, 150K, 130K, 110K, 90K) to 77K (blue). Secondary peaks/ splitting cannot be resolved at this temperature. Peak at 2.57 eV is 410 meV above the exciton peak and can be explained by coulomb enhancement at the band edge, or by a split off band. |

| 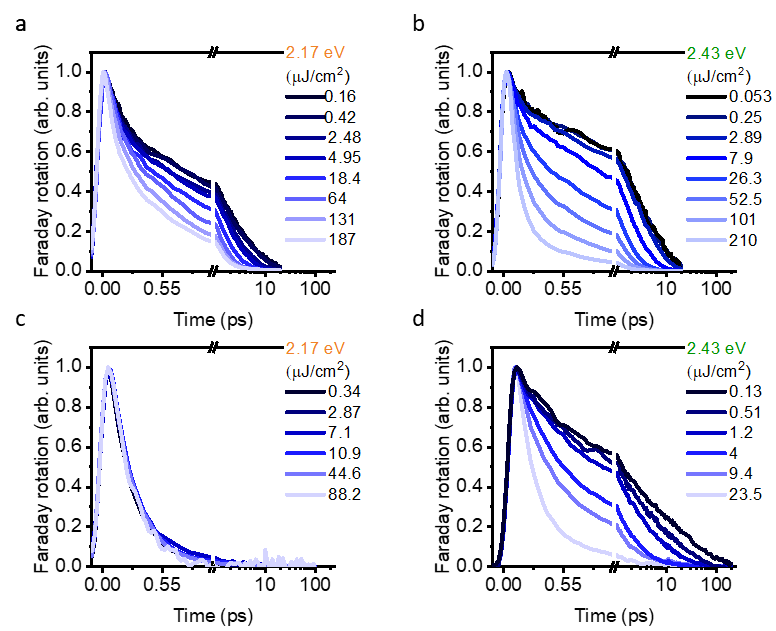 |
| --- |
| **Supplementary Figure 2: Fluence dependent Faraday Rotation measurements. a,c,** under 2.17 eV pump at room temperature and 77K respectively. **b,d,** under 2.43 eV pump at room temperature and 77K respectively. Measurements in the main text are performed with fluences below the bimolecular annihilation threshold. The exciton densities corresponding to the reported fluences are between 1.33×10^16^ and 1.55×10^19^ cm^–3^ in **a**; 0.29×10^16^ and 1.15×10^19^ cm^–3^ in **b**; 3×10^16^ and 0.79×10^18^ cm^–3^ in **c**; and 0.67×10^16^ and 1.2×10^18^ cm^–3^ in **d**. |

| 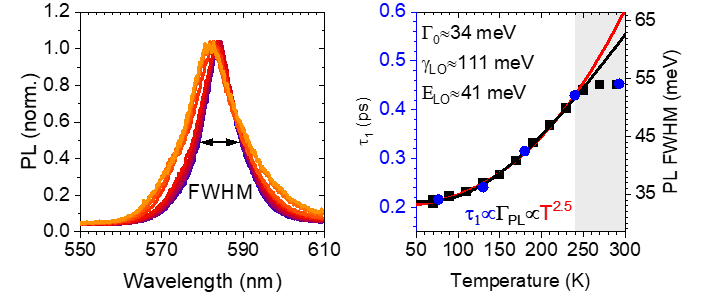  b  a |
| --- |
| **Supplementary Figure 3: Photoluminescence and spin depolarisation as a function of temperature under 2.17 eV excitation.** **a** Photoluminescence lineshape as a function of temperature from 70K (blue) through (90K, 110K, 130K, 150K, 170K, 190K, 210K, 230K, 250K, 270K) to 290K (orange) and **b**, Full-width-half-maximum (FWHM) of PL lineshape plotted as a function of temperature and compared to the (dominant) $\tau_{1}$ spin lifetime obtained from Figure 2a of the main text. |

We fit the temperature dependence of the PL linewidth with:

| $\Gamma_{\mathrm{PL}}(T)=\Gamma_{0}+\frac{\gamma_{LO}}{e^{E_{LO}/K_{B}T}-1} ,$ | **(10)** |
| --- | --- |

where $E_{LO}$ is the average energy of phonons which scatter with the exciton and $\gamma_{LO}$is the exciton-phonon coupling strength. Consistent with previous reports on a 2d perovskite variant^12,13^, this fit yields an average phonon energy of 41 meV with $\gamma_{LO}$, = 111 meV. This coupling strength is higher than typical coupling strengths found in other quantum wells^14^, confirming that strong exciton-phonon coupling exists, and is consistent with previous reports that demonstrate greater exciton-phonon coupling to a higher energy, more delocalised exciton^15^. We also observe a red-shift of the PL at low temperatures, which has been related to exciton-phonon coupling in other perovskites by previous studies^16–19^.

| 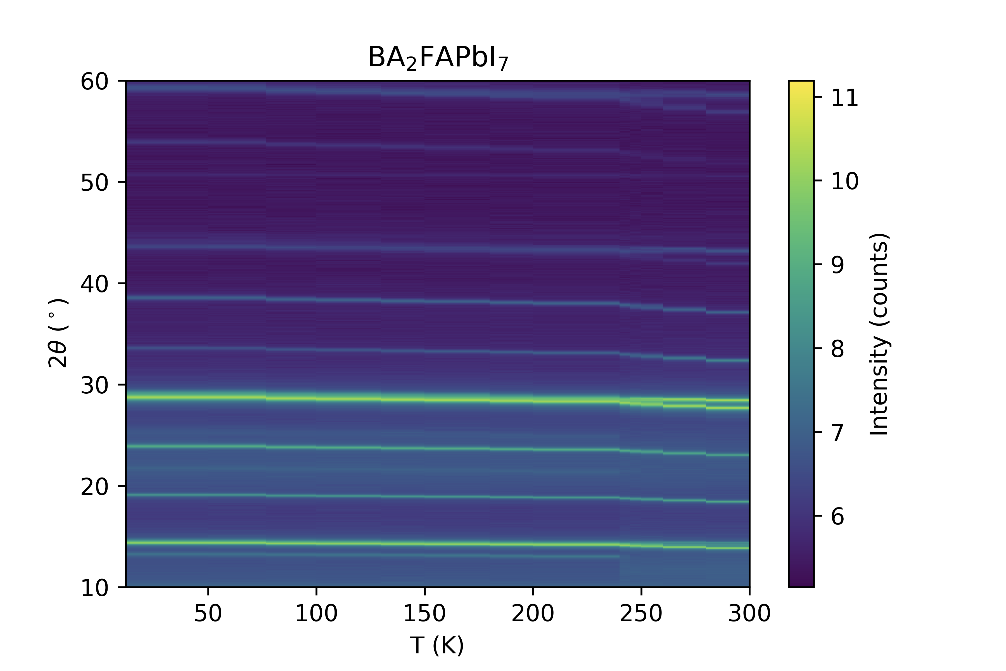 |
| --- |
| **Supplementary Figure 4:** **X-ray diffraction measurements on thin film n=2 BA_2_FAPbI_7_.** Phase transition occurs at 240K, in agreement with previous reports^5^ which identified a structural phase transition induced by alkyl chain melting. |

|  |
| --- |
| **Supplementary Figure 5:** **Spin depolarisation as a function of scattering rate, as extracted from the PL FWHM.** The linear relationship between PL FWHM and spin depolarisation time is determined from the two temperature dependent measurements, shown in the i**nset** (see also Figure 2b). PL FWHM is assumed to scale linearly with momentum scattering rate due to homogeneous broadening as discussed in the main text. |


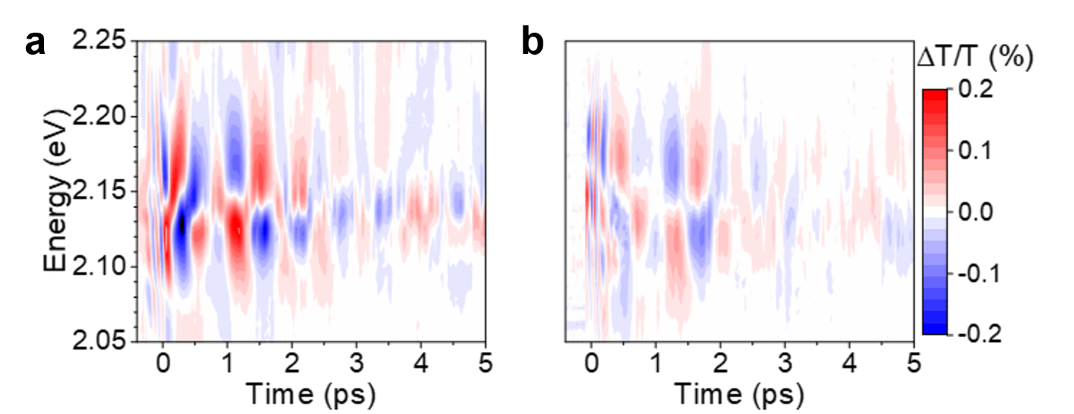


**Supplementary Figure 6: TA maps after subtraction of fit demonstrating out of phase oscillations on either side of the bleach peak**. (a) Under 2.17 eV. (b) Under 2.43 eV.


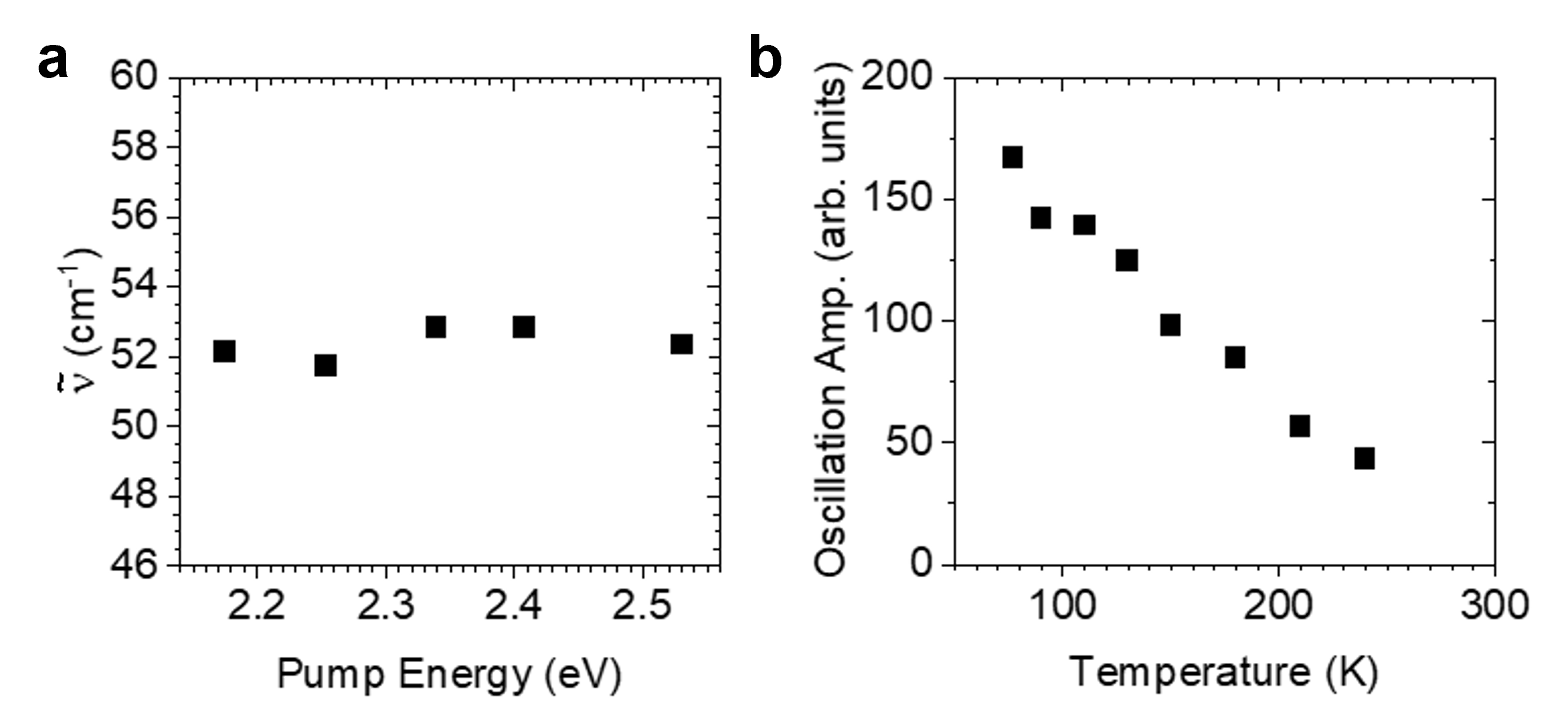


**Supplementary Figure 7: Analysis of TA oscillations under 2.17 eV pump.**(a) Oscillation frequency versus pump energy and (b) temperature dependence of the oscillation amplitude. The oscillation amplitude decreases with increasing temperature, which is expected for RISRS as the Raman band sharpens at lower temperatures^20^, but could also be explained by increasing dynamic disorder^21^.


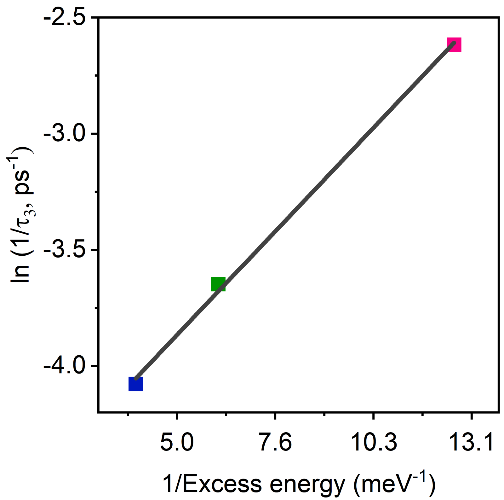


**Supplementary Figure 8: Arrhenius plot of** $\boldsymbol{\tau}_{\boldsymbol{2}}$ **as obtained in Figure 4 against the inverse excess energy provided by the excitation pulses.**Standard analysis yields an activation barrier height of 18.4 meV, which may be skewed by the lifetime inhomogeneity linked to the simultaneous character of spin relaxation and polaronic state formation. We note that 18.4 meV corresponds to the thermal energy of 210K, the temperature range for which spin lifetime changes between mono and biexponential in Figure 2b. The unusual positive slope arises from the fact that our observable is the spin lifetime, which gets slower as the activation barrier crossing gets faster.

As $\tau_{2}$ is our observable and as the starting population of excitons shows a 0.2 ps spin lifetime, we lack direct access to the transfer rate between excitons and polaronic states, since we always observe some spin relaxation while the excitons cross the activation barrier to form the polaronic states. This complicates the use of an Arrhenius plot to extract the activation barrier height. Nevertheless, we note that the spin depolarisation time $\tau_{2}$, scales with pump photon energy as it reflects the transfer rate (the ratio) between exciton and polaronic state spin depolarisation. We therefore construct an Arrhenius plot, Figure R2, which shows the expected linear behaviour with the caveat that an increase in transfer rate is observed through a *slowing* of the spin lifetime. Therefore, the resulting Arrhenius plot reflects the crossing of an activation barrier but has a very unusual positive slope.

Analysing the Arrhenius plot yields a barrier height of 18.4 meV, but this value will be skewed by the indirect nature of our observable. In any case, this value is consistent with the observation in Figure 2b that as the temperature is increased, the spin relaxation following 2.17 eV excitation stops being monoexponential at around 210K, which corresponds to a thermal energy of 18.4 meV.

**References**

1. Bourelle, S. A. *et al.* How Exciton Interactions Control Spin-Depolarization in Layered Hybrid Perovskites. *Nano Lett.* **20**, 5678–5685 (2020).

2. Giovanni, D. *et al.* Tunable room-temperature spin-selective optical Stark effect in solution-processed layered halide perovskites. *Sci. Adv.* **2**, e1600477 (2016).

3. Seitz, M. *et al.* Exciton diffusion in two-dimensional metal-halide perovskites. *Nat. Commun.* **11**, 1–8 (2020).

4. Yu, P. Y. & Cardona, M. *Fundamentals of Semiconductors: Physics and Materials Properties*. *Springer* (1999). doi:10.1007/978-3-662-03848-2.

5. Guo, Z., Wu, X., Zhu, T., Zhu, X. & Huang, L. Electron-Phonon Scattering in Atomically Thin 2D Perovskites. *ACS Nano* **10**, 9992–9998 (2016).

6. Savenije, T. J. *et al.* Thermally activated exciton dissociation and recombination control the carrier dynamics in organometal halide perovskite. *J. Phys. Chem. Lett.* **5**, 2189–2194 (2014).

7. Karakus, M. *et al.* Phonon-Electron Scattering Limits Free Charge Mobility in Methylammonium Lead Iodide Perovskites. *J. Phys. Chem. Lett.* **6**, 4991–4996 (2015).

8. Odenthal, P. *et al.* Spin-polarized exciton quantum beating in hybrid organic-inorganic perovskites. *Nat. Phys.* **13**, 894–899 (2017).

9. Sharma, R. *et al.* Elucidating the atomistic origin of anharmonicity in tetragonal CH_3_NH_3_PbI_3_ with Raman scattering. *Phys. Rev. Mater.* **4**, (2020).

10. Bakulin, A. A. *et al.* Real-Time Observation of Organic Cation Reorientation in Methylammonium Lead Iodide Perovskites. *J. Phys. Chem. Lett.* **6**, 3663–3669 (2015).

11. Duan, H. G. *et al.* Photoinduced Vibrations Drive Ultrafast Structural Distortion in Lead Halide Perovskite. *J. Am. Chem. Soc.* **142**, 16569–16578 (2020).

12. Straus, D. B. *et al.* Direct Observation of Electron-Phonon Coupling and Slow Vibrational Relaxation in Organic-Inorganic Hybrid Perovskites. *J. Am. Chem. Soc.* **138**, 13798–13801 (2016).

13. Zhang, Y. *et al.* Optical Properties of Two-Dimensional Perovskite Films of (C6H5C2H4NH3)2[PbI4] and (C6H5C2H4NH3)2(CH3NH3)2[Pb3I10]. *J. Phys. Chem. Lett.* **10**, 13–19 (2019).

14. O’Neill, M., Oestreich, M., Rühle, W. W. & Ashenford, D. E. Exciton radiative decay and homogeneous broadening in CdTe/Cd_0.85_Mn_0.15_Te multiple quantum wells. *Phys. Rev. B* **48**, 8980–8985 (1993).

15. Esmaielpour, H. *et al.* Role of Exciton Binding Energy on LO Phonon Broadening and Polaron Formation in (BA)_2_PbI_4_ Ruddlesden-Popper Films. *J. Phys. Chem. C* **124**, 9496–9505 (2020).

16. Wang, S. *et al.* Temperature-Dependent Band Gap in Two-Dimensional Perovskites: Thermal Expansion Interaction and Electron-Phonon Interaction. *J. Phys. Chem. Lett.* **10**, 2546–2553 (2019).

17. Long, H. *et al.* Exciton-phonon interaction in quasi-two dimensional layered (PEA)_2_(CsPbBr_3_)_n -1_PbBr_4_ perovskite. *Nanoscale* **11**, 21867–21871 (2019).

18. Saidi, W. A., Poncé, S. P. & Monserrat, B. Temperature Dependence of the Energy Levels of Methylammonium Lead Iodide Perovskite from First-Principles. *J. Phys. Chem. Lett* **7**, 46 (2016).

19. Johannes Hunger *et al.* Direct observation of mode-specific phonon-band gap coupling in methylammonium lead halide perovskites. *Nat. Commun.* **8**, (2017).

20. Griffiths, P. R. Introduction to Vibrational Spectroscopy. in *Handbook of Vibrational Spectroscopy* (ed. Griffiths, P. R.) (John Wiley & Sons, Ltd, 2006). doi:10.1002/0470027320.s0102.

21. Thouin, F. *et al.* Phonon coherences reveal the polaronic character of excitons in two-dimensional lead halide perovskites. *Nat. Mater.* **18**, 349–356 (2019).
